# Conservative Treatment of the Dental Pulps

**Published:** 1874-09

**Authors:** H. L. Sage


### SELECTED ARTICLES.

---

### ARTICLE IV.

### *Conservative Treatment of the Dental Pulp.*

BY H. L. SAGE, D. D. S.

This is a question which partakes of a personal nature, so far as it can be answered by a recital of personal experience in practice.

It is a fact which has come to be quite generally acknowledged, that exposed pulps can be saved in the majority of cases, and that if they *can* be, they *should* be. If they can be saved by proper treatment, it is a neglect of duty, and an act deserving of some censure to destroy a pulp, unless the conditions are such as to render an attempt to preserve vitality very much of the nature of a forlorn hope.

Granting that control of the patient can be had, even if the attempt is likely to prove a failure, the indications will usually be manifested in time to abort any serious consequences which might ensue, while, if the trial is made with due care and proper preliminary treatment when the conditions are such as to require it, it is not too much to affirm that, at least, nine-tenths of the exposed pulps which come into our hands can be protected and saved.

The day of tedious, protracted root filling, let us hope, is past, as related to teeth which have not already lost vitality or in which inflammation has not so involved the pulp as to have reached a highly congested stage. "A living dog is better than a dead lion," so a half living tooth is better than a dead one, if its presence creates no disturbance. A dead tooth has entered on a deteriorating process which will eventually cause its loss. When the pulp of a tooth dies, though the latter may for a time keep up a sort of semi vitality through its pericentum, it is liable, sooner or later, to become entirely devoid of life, when every source of nourishment having been cut off, it will either be cast aside because its presence is no longer tolerable, or hold a mere mechanical relation to the parts around it. The animal constituents, having become dead, effete and shrunken matter in the tubuli, the latter will be filled with the debris, and whatever fluids may find their way therein, either through the peripheral portion or through the tubules opening from the walls of the pulp cavity, will stagnate, and, together with the dead animal matter cause discoloration, while the integrity of its structure as related to strength will have become greatly impaired.

It may be likened to a tree which, having ceased to derive nourishment from the soil, or having been deprived from any cause of the elements of nutrition, loses the toughness of fibre which it once possessed, becomes spalt, decays, disintegrates and finally returns to the dust from which it was taken. If a tooth in which the animal matter is dead, be immersed for a few days in violet ink, for instance, the latter will pass into the tooth at the junction of the dentine and enamel, or the minute longitudinal cracks or checks in the crown, and entirely through to the centre.

What then is to prevent a dead tooth from becoming discolored more or less, according to age, density, the size of the tubules, etc.?

But this is a diversion from the subject. It shows, however, the futility of trying to make a moisture tight filling, by way of experiment, if you please, in a dead and dried extracted tooth, when fluids will penetrate the tooth from the side opposite the filled cavity, and passing entirely through it, reach and discolor the surface of the gold.

To conduct the experiment properly, the tooth should be living and fresh from the mouth, or recently living, and one in which the animal matter has not become dry. But more upon this point at a future time.

But, as bearing upon the question before us, it becomes of some importance when we enquire, what is the experience of individual practitioners with a method of treatment which has become so general, namely, the preservation of vitality in pulps which have become exposed by the encroachment of caries or by accident.

These pulps may be classified under two heads; those recently exposed and those exposed for an indefinite length of time; the latter presenting in various inflammatory conditions.

As to the sloughing or pus secreting pulps, few probably, are in the habit of treating these with a view of saving and restoring to health the feeble remnant of pulp tissue. Such a condition requires a fine degree of perceptive and manipulative talent, to diagnose and properly treat it. When pulps reach this stage, comparatively little is to be gained by conservative treatment, or only in exceptionable cases, and personally, my attention has been but little directed thereto, perhaps from some lack of faith in its practicability or importance.

In making a statement of the results of capping by the use of the oxychloride of zinc, time will not permit of entering much into details as to conditions.

Two methods were adopted, namely: capping and filling with the same material to remain a longer or shorter time. before removing a portion and protecting by a permanent filling, or, at the same sitting, capping, removing the excess and filling over with gold or amalgam, the character of the permanent filling as to material not always corresponding to the judgment of the operator, but to the pocket, economical notions, or ideas in general of the patient, as he might elect: the controlling power not always being within the influence of the dentist.

It is natural to suppose that success might not follow the first attempts to save exposed dental pulps, in so large a ratio, as after more experience had been gained by frequent cases requiring treatment.

At first, all capped pulps were allowed to run a time for trial, while now, when practicable and best, the work of capping and filling permanently is completed at same sitting, the results having seemed to show that, if the pulp is healthy, the latter is the best course that can be adopted, owing, probably, to the fact, that the fluids of the mouth will permeate an unprotected oxychloride filling, to a certain depth at least, and may thus, especially if they are unhealthy, prove irritable to the pulp.

Again, patients are not always, or indeed in most cases, watchful or prompt to return at the time appointed, but not unfrequently neglect to report until the oxychloride filling is worn away or softened at the base, and the pulp becomes again exposed or injured by contact with the fluids from without, while many times, the tooth being placed in a comfortable condition, they neglect to return at all, and it is lost by inattention.

While in some locations and in healthy mouths, oxychloride fillings will remain without deterioration for years even, in others, they will rapidly soften and wear away.

Cavities in the proximal surfaces of bicuspids and molars, if filled with this material, are, in many cases, soon left without protection, more particularly if the gums are unhealthy, and the cavity extends below the margin of the gums.

What the agent is that acts upon the filling is not certainly known, but that it is an alkali is, perhaps, more probable than that an acid produces the effect.

That ammonia is the agent is very likely. If ammonia is present in the mouth in excess, it will soften and decompose an oxychloride filling.

If a hardened mass of this material be placed for a few days in a solution of twenty drops of aqua ammonia to the ounce of water, it will become soft and chalky. If immersed in an aqueous solution of hydrochloric acid, say six drops to the ounce of water, no effect will be produced, but a strong solution of the same will dissolve the oxychloride.

Acetic acid, twenty drops to the ounce of water, has no effect, but if the acid is in the proportion of fifty drops to the ounce of water it will decompose the oxychloride. Weak aqueous solutions of chloride of sodium, caustic potassa or caustic soda are also inert if the oxychloride is placed therein. Strong aqueous solutions of caustic potassa or soda will act upon it.

Guillois' cement or oxychloride of zinc, if hardened out of the mouth, just moistened, and touched with litmus paper will present a strong acid reaction, no matter, comparatively speaking, how many times the experiment is repeated. A Guillois' cement filling which had deteriorated greatly, after a trial of a few months in a mouth in which the fluids were excessively alkaline, was washed to cleanse the surface and subjected to the same test, when it gave only very faint acid traces.

This filling was undergoing decomposition, and others which had been inserted in labial surface cavities in adjoining teeth had, from this cause, loosened and fallen out, though the fillings were at first protected thoroughly from moisture for twenty-four hours. But the gums above the cavities were unhealthy.

Litmus paper, previously reddened by an acid was held near and over them, when vapor was immediately evolved, showing the presence of a volatile alkali. Probably this was ammonia generated by the inflamed gums, though it might have been present in the breath as well, for ammonia is, according to Richardson, Viale and Latini, present in the healthy breath; taking all sources together, there may be an excess in the fluids of the mouth, in individual cases. That this was the agent that decomposed the fillings is more than likely. Ammonia is generated by decaying animal and vegetable matter. Unhealthy gums, being akin to putrefying substances, will doubtless throw off ammonia, as well as the animal and vegetable matters taken as food, if particles are allowed to remain between the teeth until decomposition sets in.

Thus we often find that oxychloride fillings, if placed in cavities extending below or above the margins of the gums, more particularly in the bicuspids and molars, are very likely to become soft and disintegrate. Here too, the gums often become unhealthy by overlapping the sharp borders of cavities, and the foreign matters which lodge therein putrefy and become poisonous and irritable to the tissues about them.

The results will then be as stated, if the gums are inflamed and the person is not particular to remove foreign matters from between the teeth, notwithstanding the filling is thoroughly protected from moisture for a day or two, as it may be by the use of the rubber dam, during the insertion of the filling, and flowing wax over it by means of a hot instrument; beside, the food is more liable to become wedged and retained between the bicuspids and molars than elsewhere, for the reason that mastication is mostly performed with these teeth and their larger proximal surfaces are more favorable for retaining it.

More might be said upon this point, and though a little off from the subject, the diversion has been natural. Now, as to capping pulps.

Since Dec. 16th, 1868, and up to the present time, I have capped two hundred and forty pulps, of which number, two hundred and four were capped previous to Jan. 1st, 1874, with the oxychloride of zinc, which is my present practice. Creosote was first applied in the majority of cases, but on May 10th, 1872 carvacrol was substituted, and has been employed since that time, to the exclusion of creosote from practice, with results fully as gratifying, and in some respects more so than when the latter was made use of. Where one employs the oxide, saturated with creosote, as a paste dressing and capping before flowing over the oxychloride, he will find it an improvement if he substitutes carvacrol for creosote; it being less irritating and more soothing to wounded or inflamed surfaces of pulp tissue.

Though carvacrol is but slightly caustic or escharotic, it penetrates tissue much more readily than creosote; and that it has more positive, certain, instantaneous and lasting effect in abating odontalgic pains than any agent in use by dentists, is a fact which practical tests have rendered clear to my mind.

The following is a synopsis of the cases treated during the time referred to, that is up to Jan. 1st, 1874:

| | | |
|---|---|---|
| Number pulps capped, | | 204 |
| Of these there were | | |
| Exposed, | 119 | |
| Exposed and bleeding, | 57 | |
| Exposed nearly, or doubtful, | 28 | |
| | | 204 |
| Temporarily filled, | 76 | |
| Permanently filled, that is, | | |
| At the same sitting, | 94 | |
| At a subsequent sitting, | 34 | |
| | | 204 |
| Of these capped pulps that produced no subsequent trouble, there were, | | |
| Tested and found to be living, | 77 | |
| Not tested, | 112 | |
| Number that produced abscess, | 4 | |
| Would not tolerate a capping, | 11 | |
| | | 204 |
| Deciduous pulps capped, | 12 | |
| Number that produced no trouble subsequently, | 12 | |

Concerning the pulps that were capped and produced no subsequent discomfort, allowance must be made for the fact that the greater number of the patients did not return to report, though many were frequently met on the street or elsewhere, while in the case of others who did return for dental operations, the fact that certain pulps had been thus treated escaped the memory of the operator, and the circumstance would not be called to mind, without referring to the record. Hence, it is fair to infer that had trouble occurred the fact would have been stated by the patient.

Beside, most of these were healthy pulps. None of the pulps that were touched with carvacrol before capping have indicated disease or produced discomfort, excepting the temporary pain which usually, though not always, follow the application of the oxychloride.

Concerning the results of recent treatment, sufficient time has not elapsed to report positive success, though pulps which will not tolerate cappings are apt to indicate it very soon in most cases. Doubtless some pulps die after capping quietly and painlessly.

Some, though living, are lame, and must be regarded as invalids to some extent—sickly, uncomplaining members of the family not quite so healthy as a pulp which has not been exposed but answering a tolerable purpose nevertheless; while with many or most healthy pulps which have been treated in this way, the situation is yet more favorable or entirely satisfactory. Hereafter, more preliminary treatment should be carefully given to exposed pulps of long standing and in various stages of inflammation, so as to be assured of their restoration to more healthy conditions, before applying the oxychloride; though it is likely that, on removing the cause of irritation and protecting by capping, the inflammation will often subside and the pulps take on healthy action without further treatment, as Case 146 may serve as an illustration.

Mr. F. M., a young man of nervo-bilious temperament presented Feb. 20th, 1873, with a cavity in the anterior proximal surface of the right inferior second bicuspid. The pulp was exposed, aching furiously, bleeding profusely and protruding from the orifice of exposure. Nothing would quiet it but carvacrol, and that not easily.

It was capped and the cavity filled with the oxychloride, the patient being instructed to return in three weeks, if no previous trouble occurred. No pain on application of the capping or during the absence of the patient, who presented Feb. 18th, 1874, ( one year thereafter, lacking two days,) for a permanent filling. The capping was very hard indeed, the pulp alive and the tooth presented every appearance of perfect health, as the sensitive margins of the cavity showed. The excess of oxychloride was removed and the layer remaining protected by a gold filling, since which time the tooth has sustained its reputation for good behavior. It would be entirely out of place to cite other examples here, of individual cases, and unnnecessary, as most or all of you concur with the view, that conservative treatment of exposed dental pulps is no longer an experiment which the results do not warrant us in continuing, but a life and health, as well as a pain and labor saving operation.—*Dental Register.*

